# The Effect of Rotational Disorder on the Microwave Transmission of Checkerboard Metal Square Arrays

**DOI:** 10.1038/srep16608

**Published:** 2015-11-16

**Authors:** B. Tremain, C. J. Durrant, I. E. Carter, A. P. Hibbins, J. R. Sambles

**Affiliations:** 1 School of Physics and Astronomy, University of Exeter, Exeter EX4 4QL.

## Abstract

The effect of rotational disorder on the microwave transmission through thin metallic checkerboard arrays has been experimentally studied. Broad resonant features below the onset of diffraction, attributed to electromagnetic radiation coupling through the structure via the evanescent fields of bound surface waves, are found to be strongly dependent on the electrical connectivity of the surface. By applying rotational disorder to the elements comprising the arrays, with the lattice constant and element size unchanged, the electrical connectivity of the structure can be controlled whilst maintaining periodicity. The results show that rotational disorder can significantly affect transmission only when it changes the structure’s connectivity. When the initial structure is just above the connectivity threshold (where the metallic occupancy is 50%), increasing disorder causes the resonant features in transmission to invert as the structure switches from a predominantly connected array to a disconnected array. When approximately half of the connections are broken, the resonant features are suppressed, with scattering loss shown to dramatically increase to as much as 40% of the incident power over a broad frequency range. The result is a thin, highly effective scatterer of microwaves.

The fabrication of thin-film structures with a specified frequency response to electromagnetic radiation has been an area of great interest in both the military and commercial sectors over the past century. Such structures have applications ranging from radome protectors in the microwave regime^1^ to solar cells in the optical^2^. These frequency selective surfaces (FSS) are often fabricated using periodic arrays of conducting patches or arrays of holes in a conducting screen^3^. In the long wavelength limit such structures act as simple inductive (hole) or capacitive (patch) meshes, however as the wavelength *λ*_0_ approaches a critical dimension of the structure, resonant surface wave effects can perturb this behaviour.

In 1998 Ebbesen *et al.* observed the enhanced transmission of electromagnetic waves in the optical regime through an array of sub-wavelength holes in a thin metallic film^4^. This enhanced optical transmission (EOT) was attributed to the excitation of surface plasmon polaritons^5^,^6^ on either side of the film that were coupled via evanescent fields within the patterned film. Ebbesen *et al.* demonstrated that transmission through such an array could exceed predictions made by Bethe’s theory for transmission through a single hole^7^ by several orders of magnitude. Following this observation there has been a substantial body of work exploring the response of sub-wavelength metallic patches and holes to optical frequency radiation^8^,^9^. A number of similar studies have been undertaken in the microwave regime exploring a range of element geometries including circular^10^,^11^, ring^12^,^13^, dipole^14^, triangular^15^ and square^16^,^17^.

One such structure is the checkerboard array consisting of a 2D square array (periodicity *λ*_g_) of perfect electric conducting (PEC) patches of side length *L,* each rotated by 45°. The ‘perfect’ checkerboard is a non-physical geometry where the corners of the squares meet at a point 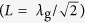. Two neighbouring patches are neither electrically connected nor disconnected and the system is at the percolation threshold. According to Babinet’s principle^18^, the transmitted intensity, *T*, through any patterned PEC sheet with no loss mechanism, is equal to the reflected intensity, 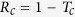, from its complementary structure (PEC areas and voids exchanged). For the self-complementary perfect checkerboard, 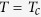, and therefore the transmitted and reflected intensity must both be equal to 1/2 for all frequencies below the onset of diffraction, resulting in an ideal beamsplitter^19^,^20^. Due to this theoretical phenomenon, the checkerboard array has received much attention. Transmission features associated with connected and disconnected checkerboard arrays have been studied experimentally in the microwave^16^, terahertz^21^, and optical^22^ regimes. Frequency-independent transmission has been observed in disordered systems where the square size is varied^21^, and by using resistive sheets to join the metallic elements^23^,^24^. In addition, a checkerboard transmission line has been used to study surface modes^25^ and layers of checkerboard arrays have resulted in a zero-index metamaterial^26^.

Strong resonant microwave transmission (reflection), similar to EOT^4^, has been observed through periodic ‘checkerboard’ square hole (patch) arrays both above and below the percolation threshold. This is attributed to bound surface waves, which are associated with coupled surface-dipole resonances on both sides of the structure. To draw a limited analogy with plasmonics, these are often termed ‘spoof surface plasmons’, but the key difference should be noted: that the character of these surface waves is not dictated by the metal’s conductivity. In connected arrays, the holes are coupled together by conventional electric current, and hence the resulting collective resonance of the holes is transverse magnetic (TM) in nature^16^. Conversely, disconnected arrays (patches) support transverse electric (TE) surface waves, with neighbouring patches interacting via displacement currents. In both cases, once excited, these surface waves can reradiate into free space. This radiation can combine constructively (or destructively) with non-resonant transmission through the structure resulting in enhanced (or reduced) transmission.

The behaviour of arrays of metallic elements close to the connectivity threshold, defined here as the critical occupancy at which metallic elements begin to overlap, is of particular importance to this study due to the fundamental difference in the nature of the bound surface waves supported by electrically connected and disconnected structures. The connectivity threshold of a variety of metamaterials has been studied by applying disorder to the arrangement of the elements comprising the array. Edmunds *et al.* explored the transmission of randomly distributed circular elements over a series of filling fractions^27^. The disruption of the array’s periodicity destroyed the resonant transmission, and the arrays showed the high and low pass filtering behaviour reminiscent of metallic meshes below the diffraction edge. Seo *et al.* reported similar findings at terahertz frequencies, demonstrating that periodic hole arrays could support surface plasmon like waves whereas similar arrays of randomly distributed holes could not^28^. The region between the periodic and the fully random has been explored by Nishiima *et al.*^29^ who illustrated the effect on plasmonic resonances of gradually increasing the spatial disorder of gold nano-disks, and reported a broadening and lowering of the transmission extinction peak. In contrast, it has been shown that strong transmission is maintained through a random array of strongly asymmetric rectangular holes^30^,^31^ and that positional disorder of a 2D array of metal-dielectric-metal stack nanoparticles has little effect on the magnetic dipole resonance^32^. Similarly, the effect of random distribution on an array of split ring resonators was shown to depend on whether the elements interact coherently or incoherently^33^. Rockstuhl *et al.* studied random arrays of circular apertures and concluded that enhanced transmission due to the resonance of a single hole acting as a waveguide was not suppressed by the presence of disorder provided a minimum hole separation was enforced such that the elements do not interact^34^. Enhanced transmission has also been reported in quasiperiodic arrays of elements. This is because the reciprocal structure (which, unlike a periodic array, does not have discrete points) has well-defined peaks^34^,^35^.

In this study, we take a similar approach to Takano *et al.* by randomly perturbing the perfect checkerboard geometry. However in this case the square size is fixed and a Gaussian random rotation is applied to each element. This allows connections between the metallic squares to be broken (or made) whilst keeping the lattice spacing constant. As the system retains this underlying periodicity, diffractive effects such as the excitation of surface waves and the onset of diffracted orders above frequency 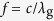 are expected to remain. We explore how disorder in element rotation of the checkerboard patch and hole arrays affects the enhanced transmission phenomena below this onset of diffraction. We begin with a square array of square metallic patches of side length *L* rotated by 45° with respect to the square lattice vectors, as shown in Fig. 1. Varying the side length of the squares changes the metal occupancy (*X*) of the array and allows transmission both below and above the connectivity threshold (*X* = 50%) to be studied. As mentioned previously, for samples of metal occupancy *X*% the transmission *T*_X_ of their inverse (or complementary) structures of metal occupancy (100 − *X*)% is 1 − *T*_X_, assuming that the only loss channels are specular reflection or transmission. This accords with the description of Babinet’s principle^18^.

Disorder in square orientation is introduced by increasing the standard deviation σ about a mean rotation μ of 0° of a Gaussian rotation distribution


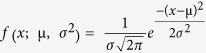


as demonstrated in Fig. 2. The standard deviation has an upper limit of approximately 26.5° due to the fact that the rotation angle is a cyclic quantity. In addition, an array with truly ‘random’ rotations is studied by applying a uniform distribution which has the same standard deviation as this upper limit.

A set of samples consisting of 300 nm thick patterned aluminium on a 50 μm thick polyester substrate were studied. The lattice spacing *λ*_g_ = 6 mm and overall sample size is 200 × 300 mm. Although the thickness of the aluminium layer is less than the skin depth (≈1 μm) in the frequency range studied, it is opaque to microwaves due to the large impedance mismatch caused by its Drude-like dielectric function. However, some electromagnetic loss to Joule heating is expected, particularly as in the thin aluminium layer there is significantly enhanced probability of collisions between free electrons and the two surfaces of the metal. Furthermore, surface waves on either side of the film will couple through the metal, not just the holes, and the existence of enhanced fields within the metal will increase power loss due to absorption. Patch arrays were created with metal occupancies *X* = 40%, 51% (just above the connectivity threshold) and 60% and 8 different standard deviations, σ (24 samples in total). In this paper we refer to each patch array by its original occupancy and the standard deviation of the patches, however note that this occupancy will change as patches begin to overlap. 24 complementary hole arrays were created by simply inverting the areas of metal and voids on the sample. For these complementary structures the occupancy refers to that of the voids, not the metal. Hence, without disorder (*σ* = 0°), a 40% patch array is identical to a 60% hole array.

## Results and Discussion

### Transmission measurements

Normal incidence transmission measurements, taken in the frequency range 26.5 ≤ *f* ≤ 60 GHz, are shown in Fig. 3. These results are based on one measurement of a single sample. Whilst there will be some variation between different samples with the same standard deviation, this variation is not significant. This is discussed further in the next section.

When σ = 0° (solid black lines) all arrays exhibit a broad resonance just below the 50 GHz onset of diffraction, manifesting as a transmission minimum for electrically disconnected arrays and a maximum for connected ones. This behaviour is much the same as the Fano-type resonant transmission observed in similar studies^16^,^17^,^18^. These resonances are due to diffractive coupling between the bound surface mode of each patch/hole array and the incident radiation. For disconnected arrays near field interactions between isolated patches are supported by displacement currents and the surface supports a TE polarized surface mode. Similarly, for connected arrays a TM mode exists, however in this case field linkage is via propagating currents. Interference between the resonant and non-resonant (straight through) transmission channels leads to the characteristic Fano-type response. A typical diffraction edge is present at 50 GHz, corresponding to the 6 mm periodicity, above which some power is lost to the first diffracted order. We restrict our discussion to lower frequencies in the non-diffractive regime. The results show that these structures do not obey Babinet’s principle, with the transmission of each patch and hole pair (left and right columns respectively) summing to approximately 80%. This violation is due to Joule losses within the metal discussed earlier. Despite the loss, the patch and hole array pairs clearly show the inverse behaviour expected and therefore the following discussion focuses on the patch array results, with the hole array results being essentially just the Babinet complement.

**Figure 1:**
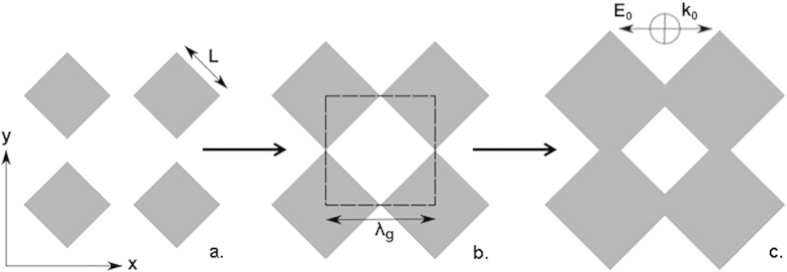
Illustration of square patches of side length L rotated at 45° to a square lattice of pitch λg = 5.95 mm. Orientation of the incident electric field vector *E*_0_ is also illustrated. (**a**) disconnected patches, *X* = 40% (**b**) threshold connection, *X* = 50% (**c**) connected patches, *X* = 60%.

**Figure 3:**
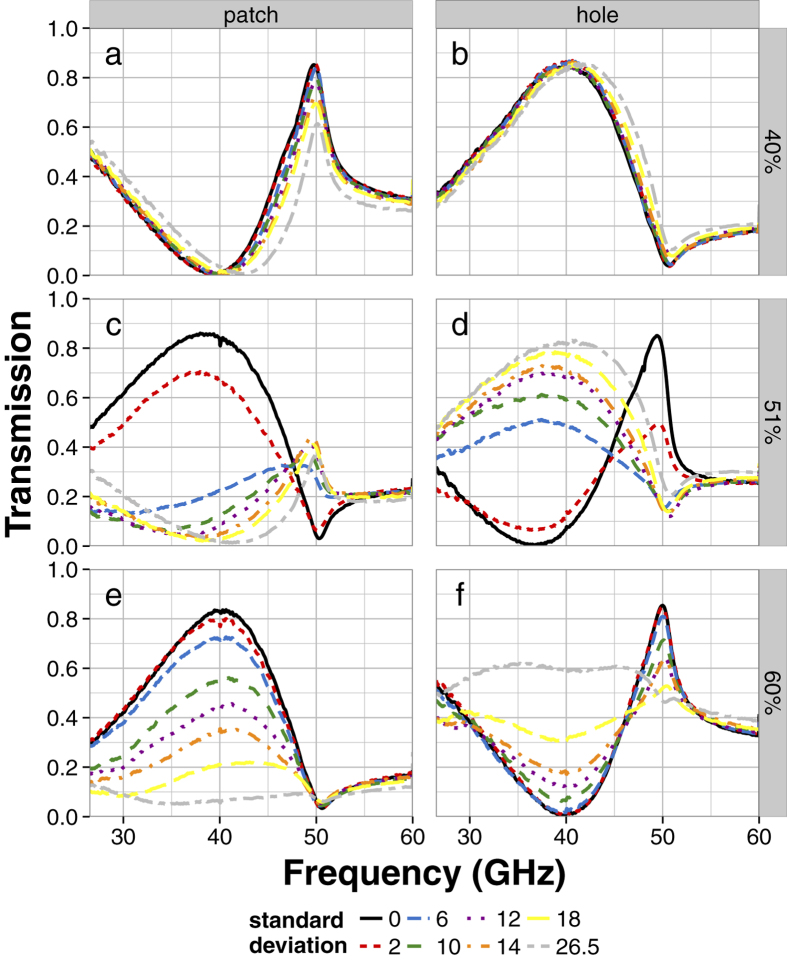
Normal incidence microwave transmission of an array of square aluminium elements rotated 45° on a square lattice λ_g_ = 5.95 mm, with increasing standard deviation in square orientation (a) 40% patch array (b) 40% hole array (c) 51% patch array (d) 51% hole array (e) 60% patch array (f) 60% hole array.

The effect of element rotation, without influence on connectivity, is illustrated by the 40% occupancy arrays in Fig. 3a,b. Below the connectivity threshold no amount of disorder can change the array connectivity. The transmission varies little with increasing disorder, highlighting that element rotation alone has little effect when it does not make or break connections between elements. These arrays show a small increase in resonant frequency with increasing σ which is attributed to the decreasing of the resonant lengths of each patch or hole in the direction of the incident polarization, *E*_0_ (Fig. 1). When σ = 0° the resonant length excited by the electric field is maximum 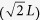. As σ is increased this varies from *L* to 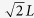. The mean value decreases leading to an increased peak frequency and a slight broadening of the resonance.

**Figure 4:**
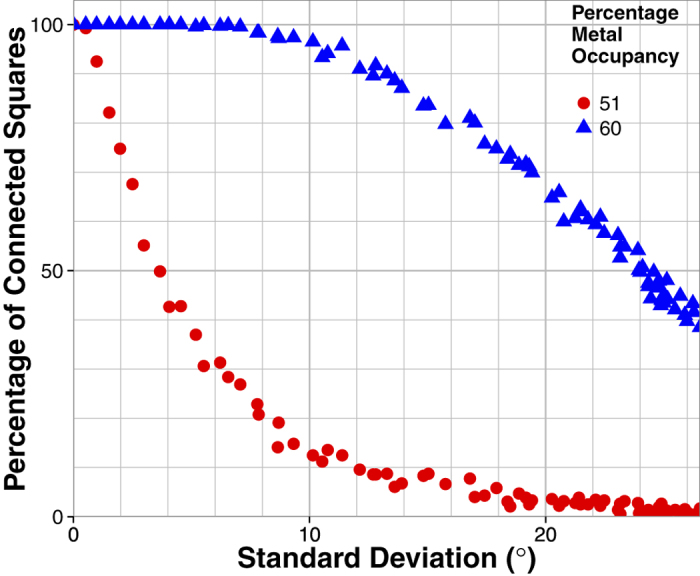
Percentage of squares that are connected to at least one neighbouring square as a function of standard deviation of the rotation for both 51% and 60% patch arrays, based on a 20 × 20 array. There is a limiting standard deviation of approximately 26.5°, which corresponds to the standard deviation of the uniformly distributed sample (here defined as ‘random’).

**Figure 5:**
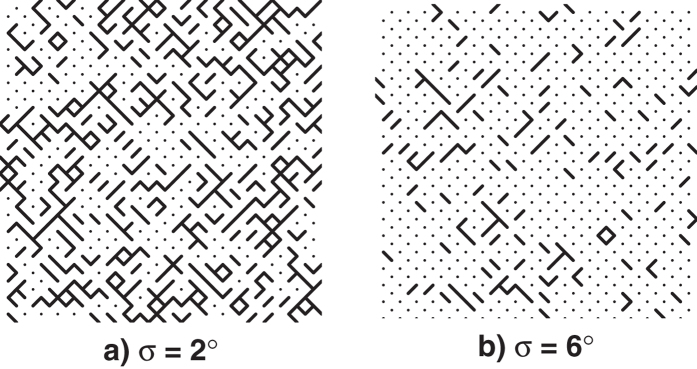
Schematics representing the difference in connectivity of the 51% patch arrays with a standard deviation of a) 2° and b) 6°. Rather than the squares themselves, connections between them are depicted as lines between the lattice points. Disconnected squares are shown as dots. At 2° disorder ~30% of squares are isolated, whereas at 6° ~ 70% are isolated.

The most interesting behaviour is observed just above the connectivity threshold in the 50% occupancy arrays. In this region the connectivity of the arrays is at its most sensitive to rotation. Figure 3c,d demonstrates strong transmission dependence on rotational disorder. For 0 ≤ σ ≤ 2° the majority of squares remain connected and so the strong resonant transmission associated with connected arrays is observed. Above this level of disorder the transmission inverts suggesting a critical change in the connectivity of the array. Figure 4 shows the percentage of square elements which are connected to at least one neighbour as a function of standard deviation. This plot was produced by analyzing 53 20 × 20 arrays each with a different standard deviation. The resonant feature is centred at 40 GHz which corresponds to a free space wavelength of approximately two connected patches. Therefore, whilst connected squares can form chains and clusters with a variety of lengths, the number of chains of at least two patches is significant for understanding the transmission inversion in Fig. 3c. For *X* = 51%, between σ = 2° and σ = 6°, Fig. 4 shows that the array promptly changes from a connected to a primarily disconnected array. This change in connectivity inverts the transmission shape as the large clusters of connected metal patches have become disconnected and so propagating currents no longer support transverse magnetic surface modes. Regions of metal patches are now linked by displacement currents, supporting a transverse electric mode and the overall transmission behaviour is that of a disconnected array. (For the 51% hole arrays which are initially disconnected, Fig. 3d, the opposite of this argument is then true as metallic regions surrounding the holes begin to connect with the introduction of disorder.) The rate of decrease of the peak transmission value with standard deviation qualitatively corresponds to the gradient of the red points within Fig. 4. The large change in peak intensity between σ = 2° and σ = 6° in Fig. 3c coincides with a large number of squares becoming isolated from their neighbours, as shown by the steep gradient in Fig. 4. The large difference in connectivity between these two arrays is demonstrated more clearly in Fig. 5. Connections between squares are shown by lines connecting the lattice points of the array with isolated squares shown as dots.

The amplitude of the resonant feature is strongly dependent on the connectivity of the structure. Before applying rotations (σ = 0°), Fig. 3c. shows a large peak due to the excited TM surface mode. This mode will propagate many wavelengths before reradiating into the specular transmission beam where it interferes with radiation that passes straight through the structure and produces a transmission peak. When the square patches are rotated such that almost all the squares are isolated from one another (σ = 18°), a TE mode is supported leading to a minimum in transmission. In between these two situations, there exists a mix of connected and disconnected regions which leads to a feature with small amplitude (between σ = 2° and σ = 6°). To understand this it is necessary to look at the reflected signal, as well as the transmission, which is discussed in the next section. The blue shift of the transmission feature arises from the fact that the longer length chains of connected squares are broken more readily by disorder than the shorter ones; hence the higher frequency connected response survives better with increasing disorder.

The 60% array is similar in that its connectivity is affected by increasing rotational disorder. However, the larger element size means low levels of disorder have a smaller effect on the transmission. The patch array shown in Fig. 3e shows a gradual decrease in resonant transmission from σ = 0° to 26.5° orientation, where the resonance is almost completely suppressed to give a flatter, approx. 5–10%, transmission response for all frequencies studied. Again, the rate of decrease in transmitted intensity correlates with the gradient of Fig. 4 (blue points). The percentage of connected squares decreases linearly above a standard deviation of 8°, as does the peak intensity in Fig. 3e. Only at σ = 26.5° does the transmission show an inversion of the resonant feature, which corresponds to approximately half of the squares being disconnected (Fig. 4). At this point, the surface wave re-radiation is small, as is the straight through transmission (due to the larger metal occupancy). The result is a tuneable transmission peak at 40 GHz which varies from 80% to 5% depending on the chosen metallic patch size.

### Scatter measurements

In order to facilitate the measurement of power lost to scatter, it is necessary to reduce the non-radiative losses (absorption) in the samples. This can be achieved by using thicker metal layers because there is a reduced probability of electron scattering with the boundary of the metal and surface waves on each interface no longer couple through the metal layer. Therefore a second set of patch samples using thicker copper with metal occupancy *X* = 51% and standard deviations *σ* = 0°, 4° (chosen as it lies at the percolation threshold, where approximately 50% of the squares are isolated, see Fig. 4), and 18° were produced. These particular values were chosen in order to ascertain how the connectivity of the structure affects scatter. In order to obtain this scatter loss both specular transmission and reflection measurements are required, together with the assumption that the absorption loss in the system is negligible.

A qualitative measure of the amount of power lost to scatter from the 51% occupancy patch arrays is obtained by adding the reflection and transmission coefficients of the thicker samples (which exhibit negligible absorption loss). In a perfect electrically conducting system, the specular reflection (R) and transmission (T) coefficients are related to the scatter loss (S) and dielectric absorption (A) by *R* + *T* = 1 − (*S* + *A*). Hence, a reduction in the sum of reflection and transmission requires an increase in either power lost to scatter (not detected in the specular transmission or reflection beam) or power lost to Joule heating in the dielectric. An increase in the latter would require greater electric field strengths within the dielectric region which would only arise if the surface waves were focussed into a particular region of the surface or if the confinement to the surface was enhanced. Neither of these effects are significant in this case and so we assume that *A* = 0 for the copper samples. We therefore use the addition of the transmission and reflection coefficients as a measure of scatter loss (1 − *S*). Experimental constraints mean that reflection measurements exactly at normal incidence are not possible and so we approximate with results at 2° to the surface normal. Whilst this small angle change will affect the shape and position of the resonant feature in reflection, the difference is negligible.

**Figure 6:**
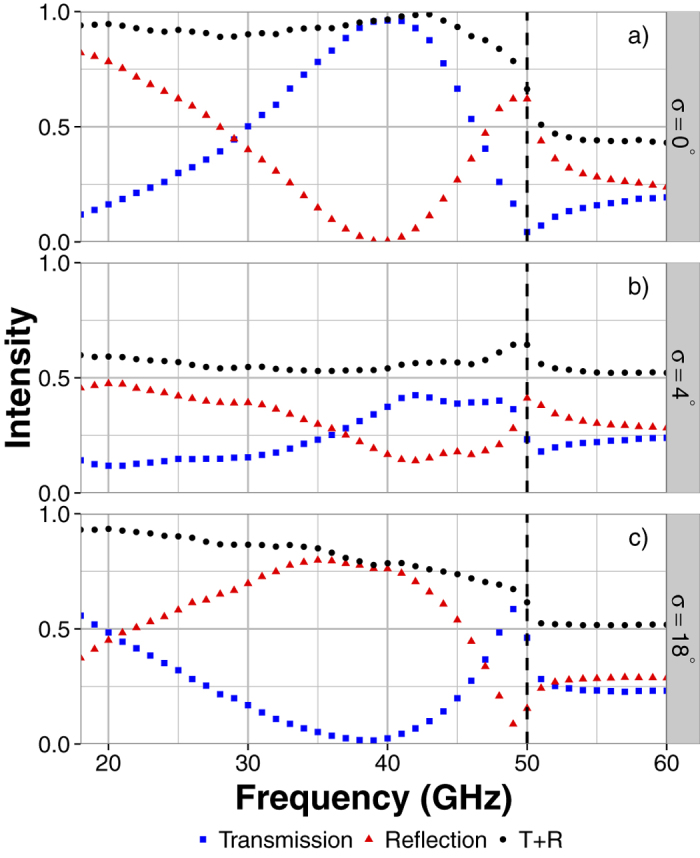
Reflected intensity (red triangles), transmitted intensity (blue squares) and their sum (black circles) for 51% occupancy copper patch arrays with standard deviations of (a) 0° (b) 4° and (c) 18°.

Figure 6 shows the reflection and transmission coefficients, as well as their sum, for square patches with 51% occupancy. The transmission coefficients (blue squares) correspond to the results in Fig. 3c, however the values are greater due to the reduced absorption loss and the frequency range is now extended. New patterns are generated with three different standard deviations; σ = 0°, 4° and 18°. For these results new samples were made using the same method but with thicker metal (18 μm copper) and larger panel size (400 × 280 mm). These changes result in much less electromagnetic loss due to absorption in the metal and scatter from the sample edges respectively. In order to verify that the incident beam is sampling a suitably large area of the sample to provide reproducible results, 6 different regions having σ = 4° have been fabricated and tested. The standard deviation of the transmitted intensities is always less than 5%, small enough that our qualitative analysis is valid. As the transmission through the σ = 4° samples is most sensitive to disorder, checking the variation of transmission for several such samples is sufficient to validate the entire data set for all other samples for which only single samples have been characterised.

Figure 6b shows that when there is a mix of disconnected and connected patches this total loss is greater than 40% of the incident power on average across the frequency range studied. This is significantly larger than the loss of 5% for σ = 0° and 20% for σ = 18° (Fig. 6a,c). This shows that, rather than propagating along the surface and reradiating into the specular reflection and transmission beams, the power coupled into the surface modes is scattered as it is unable to pass from a connected region to a disconnected one (or vice-versa) due to the mismatch in polarization. The decrease in *R* + *T* with increasing frequency for σ = 18° is attributed to the fact that there are still some small length chains of connected patches causing increased scatter at higher frequencies.

## Conclusions

A study of the effects of increasing rotational disorder on the microwave transmission response of a checkerboard square array of square metallic patches and their complementary hole arrays has been presented for a range of metal occupancies significantly above and below, as well as close to, the 50% metal occupancy connectivity threshold. This type of disorder is distinct from many ‘random’ planar filters which contain disorder in element shape, size and spacing.

It was found that disorder in the rotation of the patches had negligible effect on the transmission if it did not change the connectivity between elements of the array. However for patches (or holes) just above 50% occupancy the increase in disorder changes the connectivity of the squares and substantially weakens the strength of the resonant transmission maximum (or minimum). This is because connected and disconnected regions of the structure support surface modes with different polarizations. If the sample is not predominantly connected or disconnected, the amplitude of re-radiation from surface modes is low. The addition of reflection measurements shows that this is because the power is instead scattered away from the specular beam. Further disorder results in an inversion of the transmission feature, now a minimum, as the array becomes predominantly disconnected. This is accompanied by a reduction in scatter loss despite the array containing more disorder in element rotation.

The introduction of rotational disorder allows for control of the microwave frequency response over a wide spectral window, opening up fresh potential for Frequency Selective Surfaces. Of particular note is the ability to scatter 40% of the incident radiation away from the specular reflection and transmission beams over a 30 GHz frequency band. This control is possible due to the presence of a very broad diffractively coupled surface mode resonance supported by the checkerboard structure just below the onset of diffraction. The type of disorder chosen maintains some periodicity in the structure and hence the diffraction edge at 50 GHz is still visible even for large standard deviations in the rotation distribution.

## Methods

### Fabrication

Arrays were designed using Visual Basic scripts in CorelDraw and R scripts in RStudio. Aluminium samples were created using traditional photolithographic and chemical etching techniques to pattern a 300 nm thick layer. Thicker samples were fabricated via a ‘print and etch’ method^36^ and consist of an 18 μm copper layer. Both Al and Cu samples are supported on a 50 μm thick polyester substrate (permittivity ε ≈ 3.2 + i0.01 at 10 GHz).

### Transmission experiment

Microwave radiation is emitted from narrow band horns connected to an Agilent E8247C signal generator. The 3 frequency bands used are 18–26.5 GHz, 26.5–40 GHz and 40–60 GHz. A 1 m focal length parabolic mirror collimates the radiation into an approximately 20 cm diameter beam which passes through the sample at normal incidence. Another mirror focusses the transmitted beam into a second horn connected to an Agilent 8757D Scalar Network Analyzer. The resulting spectra are normalized to the transmission with no sample.

### Reflection experiment

Reflection measurements are obtained using one mirror to simultaneously collimate the incident radiation and focus the reflected beam. The emitting and receiving horns are placed adjacent to each other (separated by approximately 3 cm), illuminating the sample at ≈2°. Results are normalized to the reflection from a planar mirror.

## Additional Information

**How to cite this article**: Tremain, B. *et al.* The Effect of Rotational Disorder on the Microwave Transmission of Checkerboard Metal Square Arrays. *Sci. Rep.* **5**, 16608; doi: 10.1038/srep16608 (2015).

## Figures and Tables

**Figure 2:**
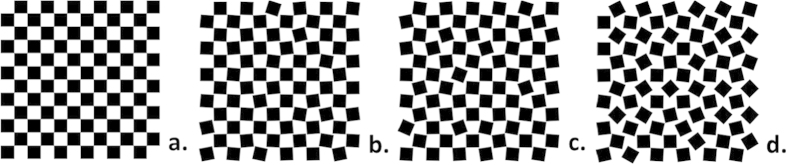
50% metal occupancy arrays with increasing rotational randomness in square orientation defined by an increase in the standard deviation (σ) of a Gaussian distributed rotation: (a) σ = 0° (b) σ = 6° c) σ = 10° (d) random (uniform distribution corresponding to σ = 26.5°).

## References

[b1] Ngai E. C. “Electromagnetic properties of metal space frame radomes for use in satellite communication earth stations”. Antennas and Propagation Society International Symposium 1993. AP-S Digest **3**, 1956–9 (1993).

[b2] Pillai S., Catchpole K. R., Trupke T. & Green M. A. “Surface plasmon enhanced silicon solar cells”. J. Appl. Phys. 101, 093105 (2007).

[b3] Mittra R., Chan C. H. & Cwik T. “Techniques for analyzing frequency selective surfaces – a review”. P. IEEE 76, 1593–1615 (1988).

[b4] Ebbesen T. W., Lezec H. J., Ghaemi H. F., Thio T. & Wolf P. A. “Extraordinary optical transmission through sub-wavelength hole arrays”. Nature 391, 667–669 (1998).

[b5] Ghaemi H. F., Thio T., Grupp D. E., Ebbesen T. W. & Lezec H. J. “Surface plasmons enhance optical transmission through subwavelength holes”. Phys. Rev. B. 58, 6779 (1998).

[b6] Popov E., Neviere M., Enoch S. & Reinisch R. “Theory of light transmission through subwavelength periodic hole arrays”. Phys. Rev. B. 62, 16100 (2000).

[b7] Bethe E. “Theory of diffraction by small holes”. Phys. Rev. 66, 163 (1944).

[b8] Genet C. & Ebbesen T. W. “Light in tiny holes”. Nature 445, 39 (2007). 17203054 10.1038/nature05350

[b9] Martín-Morenon L. *et al.* “Theory of extraordinary optical transmission through subwavelength hole arrays”. Phys. Rev. Lett. 86, 6 (2001). 11178023 10.1103/PhysRevLett.86.1114

[b10] Pous R. “A frequency selective surface using aperture coupled microstrip patches”. IEEE T. Antenn. Propag. 39, 1763 (1991).

[b11] Lockyer D. S. & Simpkin R. A. “Complementary frequency selective surfaces”. IEE P –Microw. Anten. P. 147, 501 (2000).

[b12] Parker E. A. & Hamdy S. M. A. “Rings as elements for frequency selective surfaces”. Electron. Lett. 17, 612 (1991).

[b13] Parker E. A., Hamdy S. M. A. & Langley R. J. “Arrays of concentric rings as frequency selective surfaces”. Electron. Lett. 17, 880 (1981).

[b14] Parker E. A. & El Sheikh A. N. A. “Convoluted dipole array elements”. Electron. Lett. 27, 322 (1991).

[b15] Stevens G., Edmunds J. D., Hibbins A. P. & Sambles J. R. “Microwave transmission of a hexagonal array of triangular metal patches”. Prog. Electromagn. Res. 20, 219 (2011).

[b16] Edmunds J. D., Taylor M. C., Hibbins A. P., Sambles J. R. & Youngs I. J. “Babinet’s principle and the band structure of surface waves on patterned metal arrays”. J. Appl. Phys. 107, 103108 (2010).

[b17] Taylor M. C., Hibbins A. P. & Sambles J. R. “Electromagnetic response of closely spaced metal meshes”. Phys. Rev. B. 86, 35126 (2012).

[b18] Booker H. G. “Slot aerials and their relation to complementary wire aerials (Babinet’s principle)”. Journal of the Institution of Electrical Engineers—Part IIIA: Radiolocation. 93, 620 (1946).

[b19] Compton R. C. “Babinet’s principle applied to ideal beam-splitters for submillimetre waves.“ Opt. Acta Int. J. Opt. 31, 515–524 (1984).

[b20] Kempa K. “Percolation effects in the checkerboard Babinet series of metamaterial structures”. Phys. Status Solidi—Rapid Res. Lett. 4, 218–220 (2010).

[b21] Takano K. *et al.* “Crossover from capacitive to inductive electromagnetic responses in near self- complementary metallic checkerboard patterns”. Sci. Technol. 515, 1222–1225 (2010). 10.1364/OE.22.024787 25322053

[b22] Ramakrishna S. A. *et al.* “Plasmonic interaction of visible light with gold nanoscale checkerboards”. Phys. Rev. B - Condens. Matter Mater. Phys. 84, 1–11 (2011).

[b23] Nakata Y., Urade Y., Nakanishi T. & Kitano M. “Plane-wave scattering by self-complementary metasurfaces in terms of electromagnetic duality and Babinet’s principle”. Phys. Rev. B 88, 205138 (2013).

[b24] Urade Y., Nakata Y., Nakanishi T. & Kitano M. “Frequency-independent response of self-complementary checkerboard screens”. Phys. Rev. Lett. 114, 237401 (2015). 26196824 10.1103/PhysRevLett.114.237401

[b25] González-Ovejero D. *et al.* “Basic properties of checkerboard metasurfaces”. IEEE. Antenn. Wirel. PR 14, 406–409 (2015).

[b26] Kwon D.-H., Li L., Bossard J. A., Bray M. G. & Werner D. H. “Zero index metamaterials with checkerboard structure”. Electronics Letters 43, 319 (2007).

[b27] Edmunds J. D. “Microwave transmissivity of sub-wavelength metallic structures”. PhD thesis, University of Exeter (2011).

[b28] Seo M. A. *et al.* “Enhanced terahertz transmission: comparisons between metal and absorber, random and periodic arrays of holes”. Conference on Lasers and Electro-Optics, and Quantum Electronics and Laser Science Conference CLEO/QELS (2006).

[b29] Nishijima Y., Rosa L. & Juodkazis S. “Surface plasmon resonances in periodic and random patterns of gold nano-disks for broadband light harvesting”. Opt. Express. 20, 11466 (2012). 22565766 10.1364/OE.20.011466

[b30] Lee J. *et al.* “Terahertz electromagnetic wave transmission through random arrays of single rectangular holes and slits in thin metallic sheets”. Phys. Rev. Lett. 99, 1–4 (2007). 10.1103/PhysRevLett.99.137401 17930634

[b31] Ruan Z. & Qiu M. “Enhanced transmission through periodic arrays of subwavelength holes: the role of localized waveguide resonances”. Phys. Rev. Lett. 96, 233901 (2006). 16803379 10.1103/PhysRevLett.96.233901

[b32] Albooyeh M. *et al.* “Resonant metasurfaces at oblique incidence: interplay of order and disorder”. Sci. Rep. 4, 4484 (2014). 24670919 10.1038/srep04484 PMC3967200

[b33] Singh R., Lu X., Gu J., Tian Z. & Zhang W. “Random terahertz metamaterials”. J. Opt. 12, 015101 (2010).

[b34] Rockstuhl C., Lederer F., Zentgraf T. & Giessen H. “Enhanced transmission of periodic, quasiperiodic, and random nanoaperture arrays”. Appl. Phys. Lett. 91, 151109 (2007).

[b35] Przybilla F., Genet C. & Ebbesen T. W. “Enhanced transmission through penrose subwavelength hole arrays”. Appl. Phys. Lett. 89, 121115 (2006).

[b36] Hooper I. R., Tremain B., Dockrey J. A. & Hibbins A. P. “Massively sub-wavelength guiding of electromagnetic waves”. Sci. Rep. 4, 7495 (2014). 25510662 10.1038/srep07495 PMC5378945

